# CD64 plays a key role in diabetic wound healing

**DOI:** 10.3389/fimmu.2024.1322256

**Published:** 2024-03-08

**Authors:** Xiuqin Zhang, Liuhong Yuan, Zhenyu Tan, Huiyan Wu, Feier Chen, Junjie Huang, Pengjun Wang, Brett D. Hambly, Shisan Bao, Kun Tao

**Affiliations:** ^1^Department of Pathology, Tongji Hospital, School of Medicine, Tongji University, Shanghai, China; ^2^Department of Pathology, Tongren Hospital, Shanghai Jiaotong University School of Medicine, Shanghai, China

**Keywords:** diabetes mellitus, CD64, CD163^+^ M2 macrophage, wound healing, CD68/CD80 M1 macrophages

## Abstract

**Introduction:**

Wound healing poses a clinical challenge in diabetes mellitus (DM) due to compromised host immunity. CD64, an IgG-binding Fcgr1 receptor, acts as a pro-inflammatory mediator. While its presence has been identified in various inflammatory diseases, its specific role in wound healing, especially in DM, remains unclear.

**Objectives:**

We aimed to investigate the involvement of CD64 in diabetic wound healing using a DM animal model with CD64 KO mice.

**Methods:**

First, we compared CD64 expression in chronic skin ulcers from human DM and non-DM skin. Then, we monitored wound healing in a DM mouse model over 10 days, with or without CD64 KO, using macroscopic and microscopic observations, as well as immunohistochemistry.

**Results:**

CD64 expression was significantly upregulated (1.25-fold) in chronic ulcerative skin from DM patients compared to non-DM individuals. Clinical observations were consistent with animal model findings, showing a significant delay in wound healing, particularly by day 7, in CD64 KO mice compared to WT mice. Additionally, infiltrating CD163^+^ M2 macrophages in the wounds of DM mice decreased significantly compared to non-DM mice over time. Delayed wound healing in DM CD64 KO mice correlated with the presence of inflammatory mediators.

**Conclusion:**

CD64 seems to play a crucial role in wound healing, especially in DM conditions, where it is associated with CD163^+^ M2 macrophage infiltration. These data suggest that CD64 relies on host immunity during the wound healing process. Such data may provide useful information for both basic scientists and clinicians to deal with diabetic chronic wound healing.

## Introduction

Compromised wound healing presents a considerable challenge, especially for immunocompromised individuals, causing substantial physical and psychological burdens worldwide (1). Among the various factors contributing to delayed wound healing, diabetes mellitus (DM) is recognized for its detrimental effects on the process. This is primarily attributed to compromised host immunity, often leading to prolonged wound healing and, in severe cases, potential amputation in susceptible patients (2).

The wound healing process is intricate, encompassing four overlapping phases: coagulation, inflammation, proliferation, and remodeling (3). Various immune cells and both pro- and anti-inflammatory mediators play pivotal roles in this process (3). Studies have documented elevated levels of pro-inflammatory mediators such as IL-1β (4), TNF (5, 6), IL-23 (7), IL-27 (8), alongside anti-inflammatory mediators including TGFβ1 (9), IL-10 (10), IL-35 (11). In previous research, we established that wound healing is significantly impaired in the context of diabetes mellitus (DM), with GM-CSF-regulated macrophages playing a mediating role (12). Specifically, during the inflammatory phase of diabetic chronic wound healing, immune cell dysfunction, notably involving a subset of macrophages known as M2 macrophages, has been shown to be crucial (13). Therefore, comprehending these immune-related mechanisms is vital for the development of effective strategies to enhance wound healing outcomes not only in diabetic patients but also in other immunocompromised individuals.

CD64, also known as IgG Fc segment receptor 1 (Fcgr1), belongs to the immunoglobulin superfamily and holds a pivotal role in the immune response, by recognizing immunoglobulins and binding to IgG (14). CD64 is predominantly expressed on myeloid cells, including monocytes, macrophages, neutrophils, and dendritic cells (15). Previous studies have indicated that CD64 plays a significant role in neutrophil recruitment during acute infectious diseases (16). Furthermore, there is evidence suggesting its involvement in certain chronic autoimmune conditions such as systemic lupus erythematosus (SLE), rheumatoid arthritis (RA), and atopic dermatitis (17–19).

However, the precise role of CD64 in wound healing among DM individuals remains uncertain. In the present study, our objective was to explore the connection between CD64 and wound healing in both DM patients and an animal model. These findings may offer valuable insights into the mechanisms underlying chronic ulceration in DM patients and contribute to a better understanding of this intricate process.

## Materials and methods

### Clinical cases

Demographic information was obtained from the electronic database of Tongren Hospital, including 56 patients with type II DM who underwent amputation due to diabetic gangrene and 10 patients who underwent surgical procedures for non-DM (**Table 1**). Skin were collected from these patients and sent to the Department of Pathology for routine histopathological confirmation.

**Table 1:** Clinical information of skin cases in diabetic and non-diabetic groups.

Variable	Diabetes Mellitus n=56	Control n=10	P-value
**Sex, male:**	42 (75.0)^a^	8 (80.0)^a^	1.000
**Age, years:** **<45** **≥45-<65** **≥65**	63.5 (58.0, 71.0)^b^ 2 28 26	56.5 (44.5, 69.0) ^b^ 2 5 3	0.124
**BMI, kg/m²:** **<25** **≥25-<30** **≥30**	23.3 (20.8, 25.9) ^b^ 40 13 3	23.8 (20.3, 24.9) ^b^ 8 2 0	0.892
**Systolic pressure, mmHg:** **<140** **≥140**	142.790 ± 2.720^c^ 34 22	127.000 ± 5.461 ^c^ 8 2	0.017
**Duration of diabetes, years:** **<5** **≥5-<10** **≥10**	10.870 ± 1.150 17 7 32	/	/
**FBG, mmol/L:** **<7** **≥7-<11.1** **≥11.1**	9.720 ± 0.439 ^c^ 12 27 17	5.140 ± 0.502 ^c^ 10 0 0	<0.001
**HbA1c, %:**	8.835 ± 2.223 ^c^	/	/
**GA, %:**	25.250 ± 7.771 ^c^	/	/

BMI, body mass index; FBG, fasting blood glucose; HbA1c, hemoglobin A1c; GA, glycated serum albumin. ^a^Data are the numbers (percentage). ^b^Data are the medians (interquartile range). ^c^Data are the means ± standard deviations.

### Tissue array, histology and immunohistology of patients

Paraffin skin samples were collected from the Department of Pathology at Shanghai Tongren Hospital, China, between 2017 and 2022. The study received approval from the Ethics Committee of Tongren Hospital, Shanghai Jiaotong University School of Medicine. Informed consent was obtained from each patient prior to surgery. From each specimen, relatively normal skin tissue was carefully selected from the wax blocks to create tissue chips containing 4 x 5 cores of 5 mm each. The blocks were sectioned at 5 µm for HE staining, as described (20).

Immunohistochemistry was performed as described in the relevant reference. The primary antibody against CD64 with ab203349 at 1/800 dilution (Abcam, Cambridge, UK) was used to detect CD64 expression. The secondary antibody was provided by BOND Polymer Refine Detection (Leica Biosystems Newcastle Ltd), and the color development was performed according to the manufacturer’s instructions. CD64 expression levels were measured using the HALO image analysis platform in a double-blind fashion for tissues from both DM and non-DM patients (21, 22).

### Construction of CD64 knockout mice

CD64 knockout (KO) mice were provided by Shanghai Model Organism (Shanghai, China) (https://www.modelorg.us/?lang=en-us). For easy understanding the CD64 knockout approach, a schematic figure was presented (**Supplementary Figure S1**). DNA fragments that contain a 567 bp sequence in Wt mice and an ≈1000 bp sequence in CD64 KO mice were amplified by PCR using the primers listed in **Supplementary Table S1**. PCR confirmation of CD64 KO mice was depicted in **Supplementary Figure S2**.

### Induction of diabetic mice

Male, 14 week old CD64 KO and Wt mice on a C57 background were procured from Shanghai Model Organism, Shanghai, China (https://www.modelorg.us/?lang=en-us). The CD64 KO mice were validated by PCR (**Supplementary Figure S2**). The mice were housed in groups of four per cage under SPF conditions, with a 12-hour light/dark cycle *ad libitum*. All experimental procedures were conducted in accordance with the guidelines approved by the Institutional Laboratory Animal Care and Use Committee.

To induce diabetes, both CD64 KO and WT mice were administered STZ (Sigma, U.S.A.). Multiple low doses of STZ (40 mg/kg/d, i.p.) in 50 mM sodium citrate buffer (pH 4.5) on five consecutive days, as described while non-diabetic mice received vehicle only (23). Each set of experiments involved a group of 16 mice. Daily recordings of body weights were collected throughout the study. Diabetic status was confirmed after 9 days through tail vein blood glucose testing using an automated Accu-Chek glucometer. Mice with non-fasting blood glucose levels exceeding 11.1 mmol/L on two consecutive non-concurrent days were considered diabetic (12, 23), whereas those with lower values were excluded from the study. The maintenance of a diabetic state was confirmed by weekly tail vein blood glucose measurements.

### Wound protocol

The backs of the animals were carefully shaved and then swabbed with 70% alcohol after anesthetic. Full-thickness skin wounds (1.0 x 1.0 cm) were created in a sterile manner and left unsutured without any dressing, following the method described in (12). To prevent traumatic injury caused by other mice, all animals were housed individually after the surgery. Skin samples were collected at specific time points: days 1, 3, 7, and 14 post-wounding, which included a 4-mm margin of unwounded skin around each wound. Each specimen was fixed in Histochoice to enable subsequent histopathological analyses.

### Wound healing analysis

Digital photographs of the skin wound sites in each animal, along with a scale bar, were taken at various time points: days 0, 1, 3, 5, 7, 10, and 14 post-wounding. The assessment of wound closure was performed in a double-blind manner using Image J software. The wound areas were standardized by comparing them with the original wound size and expressed as a percentage of wound closure using the formula: [(day 0 area) - (day n area)]/(day 0 area) x 100. The data were presented as mean ± standard deviation (SD) and subjected to multiple comparisons using one-way analysis of variance (ANOVA) to analyze differences among different groups.

### Histology, immunohistology and immunofluorescence of mice

Tissues were sectioned at a thickness of 5 µm and subjected to both HE staining and immunohistochemical staining following specified procedures. The antibodies utilized for immunohistochemistry were sourced from AbCam, based in Cambridge, UK. Detailed information, including catalog numbers and dilutions for each antibody, is explicitly provided, such as CD64 (ab203349, 1/800), CD163 (ab182422, 1/800), CD31 (ab281583, 1/8000), and TGFβ1 (ab215715, 1/250).

The procedures for immunohistological staining and the specific antibodies used were previously described in detail (12, 24, 25). TGFβ1 labelling is performed as described previously (26). Representative images are provided in accordance with the details outlined.

The numbers of CD163^+^ macrophages within the wound beds were quantified using the HALO image analysis platform (22). Specifically, 12 randomly chosen visual fields (at 400x magnification) of each mouse’s section, stained with the anti-CD163 antibody, were analyzed, and the mean count was calculated. Similarly, CD31 staining was assessed using Image J in the same manner, with 12 randomly selected visual fields (at 400x magnification) of each mouse’s section, and the mean count was determined. For both CD163^+^ and CD31 measurements, all analyses were performed under double-blind conditions to minimize bias and ensure unbiased results.

**Figure 1:**
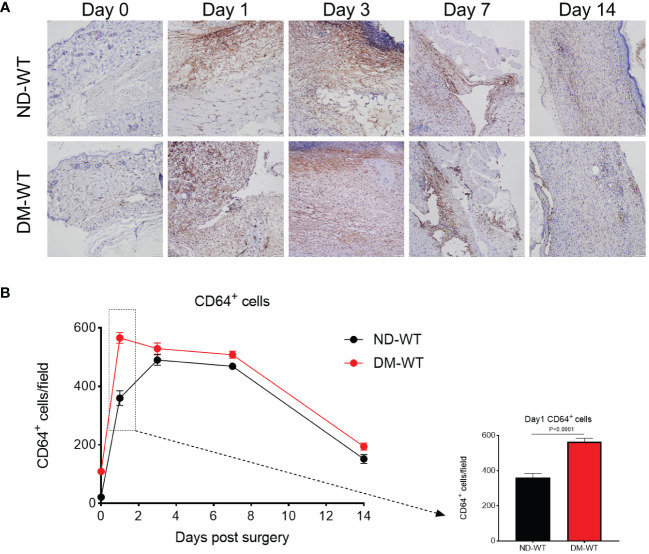
CD64^+^ cells in the wounds across different groups from Wt. The representing photos are presented in **(A)**, while the quantification is presented in **(B)**. The X-axis denotes the days post-surgery, while the Y-axis represents the number of CD64^+^ cells per high power field.

Immunohistochemistry was conducted to identify CD64^+^ cells, and the quantification of these positive cells per field was performed using HALO image analysis platform (22). The kinetics of CD64^+^ cells in wounds from Wt mice are depicted in **Figure 1**. The corresponding images are presented accordingly.

Immunofluorescence double staining was utilized to assess the expression of macrophages. Tissue sections were incubated with specific antibodies, including anti-F4/80 (ab111101, 1/250, Abcam, Cambridge, UK), anti-CD80 (ab254579, 1/500, Abcam, Cambridge, UK), and anti-CD163 (ab182422, 1/500, Abcam, Cambridge, UK), overnight at 4°C. Following this, the sections were treated with the appropriate fluorescent secondary antibodies: anti-F4/80 antibody paired with its counterpart from Beijing Panovue Biological Technology, China, and CD80 or CD163 with their respective fluorescent secondary antibodies from Beijing Panovue Biological Technology, China. Nuclei were identified using DAPI (Beijing Panovue Biological Technology, China). Co-localization was visualized using a BX60 Olympus fluorescence microscope, and quantitative analysis was conducted using Halo digital imaging analysis software 2.0 (Indica Labs, USA).

Trichrome staining was used for collagen identification. The ratio of collagen-positive blue area to that of the total tissue area was calculated by collagen volume fraction (27), using the computer-imaging software Image J. For each section ten horizontal photographs were randomly (× 400) evaluated, whereby collagen volume fraction = (collagen area)/(full tissue area), and taking the average.

The experiment has been approved by Human and Animal Ethic Committee, Shanghai Tongren Hospital.

### Statistical analyses

Statistical analyses were conducted using GraphPad Prism version 8 (GraphPad Software, San Diego, CA, United States). One-way analysis of variance (ANOVA) followed by Tukey’s multiple comparison test was employed to compare group means for CD64^+^ cells of patients. Two-way ANOVA was used to compare group means followed by Holm-Sidak *post-hoc* test for CD64^+^ cells of mice. Three-way ANOVA followed by Holm-Sidak *post-hoc* test was conducted for group mean comparisons related to wound closure, CD163^+^ macrophages, TGF-β^+^ cells, CD31^+^ cells of mice. Clinical information, such as age, sex, and BMI, was tested using the Mann–Whitney U-test and Pearson’s chi-squared test. A significance level of P < 0.05 was considered statistically significant.

## Results

### CD64 was closely associated with diabetic wound skin

CD64 expression in mice skin from DM-WT group was higher than in ND-WT tissues (**Figure 1**). Following surgery, CD64 expression in the diabetic group increased and peaked at day 1, whereas the expression of CD64 in the non-diabetic group reached its peak on the 3^rd^ day. The difference between DM-WT and ND-WT was most pronounced on day 1: CD64 expression in skin from DM mice was approximately 1.5-fold higher than in non-DM tissues (P<0.001). There was no detectable CD64 from ko mice, as expected and confirmed by the commercial supplier.

**Figure 2:**
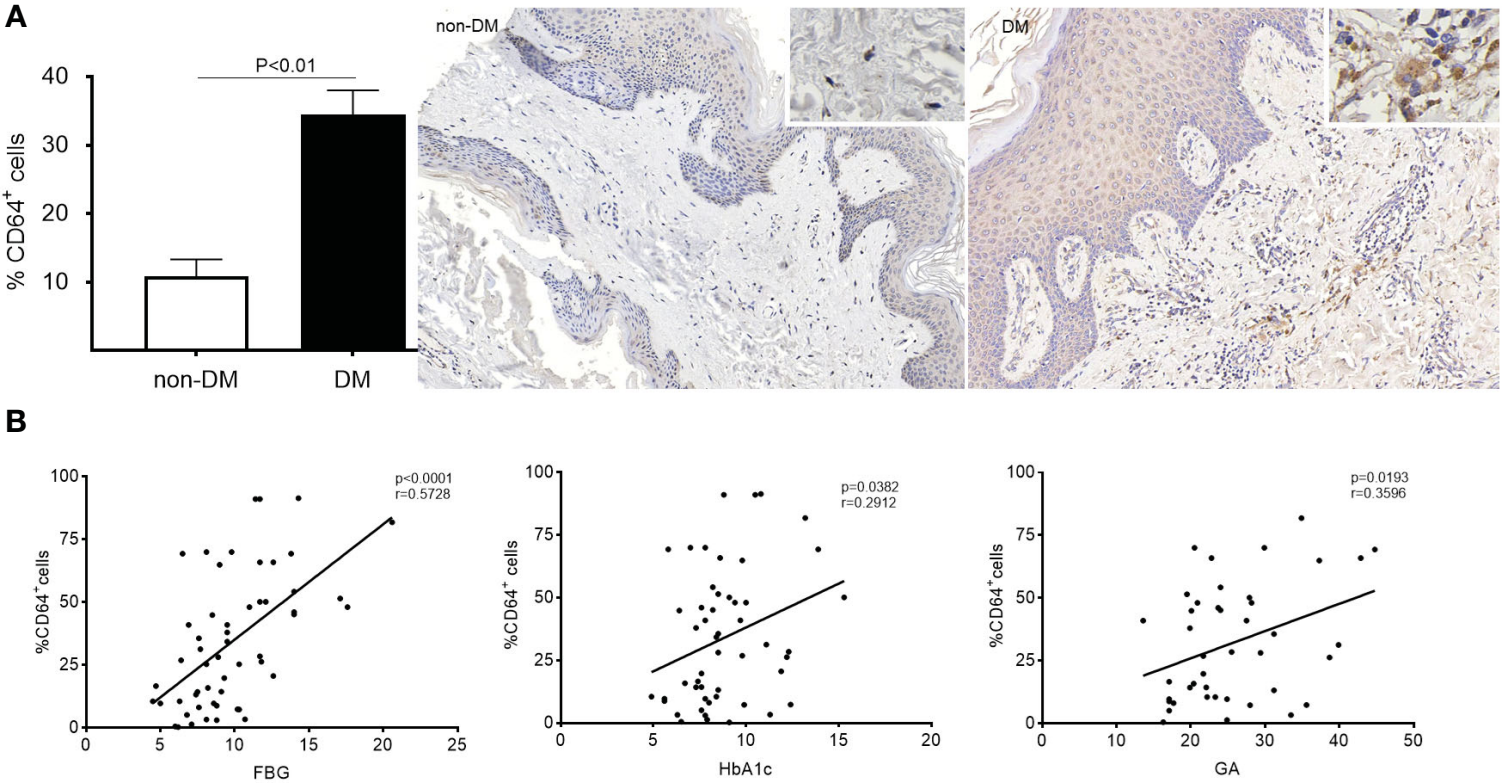
Expression of CD64 in diabetic and non-diabetic skin detected by immunohistochemistry. Data are presented as mean ± standard deviation in the left graph **(A)**. The correlation between %CD64^+^ cells and FBG, HbA1c and GA was analysed **(B)**.

The patients with diabetic foot were predominantly male, with a majority being elderly (almost half of them aged >65). Their fasting blood glucose (FBG) levels were mostly elevated (> 7 mmol/L), along with high levels of HbA1c (mean = 8.835%) and GA (mean = 25.25%). In both DM and non-DM patients, CD64 expression was mainly observed in the dermal layer of the skin (**Figure 2A**). Remarkably, CD64 expression in skin from DM patients was approximately 3-fold higher than in non-DM tissues. Furthermore, a significant correlation was observed between CD64 expression and FBG (P<0.001), CD64 expression and GA (P<0.05), as well as CD64 expression and HbA1c (P<0.05) in diabetic patients (**Figure 2B**). These findings indicate the potential relevance of CD64 in diabetic wound healing and its association with glycemic control markers in diabetic patients.

### CD64 gene knockout delayed wound healing in diabetic and non-diabetic skin

**Figure 3:**
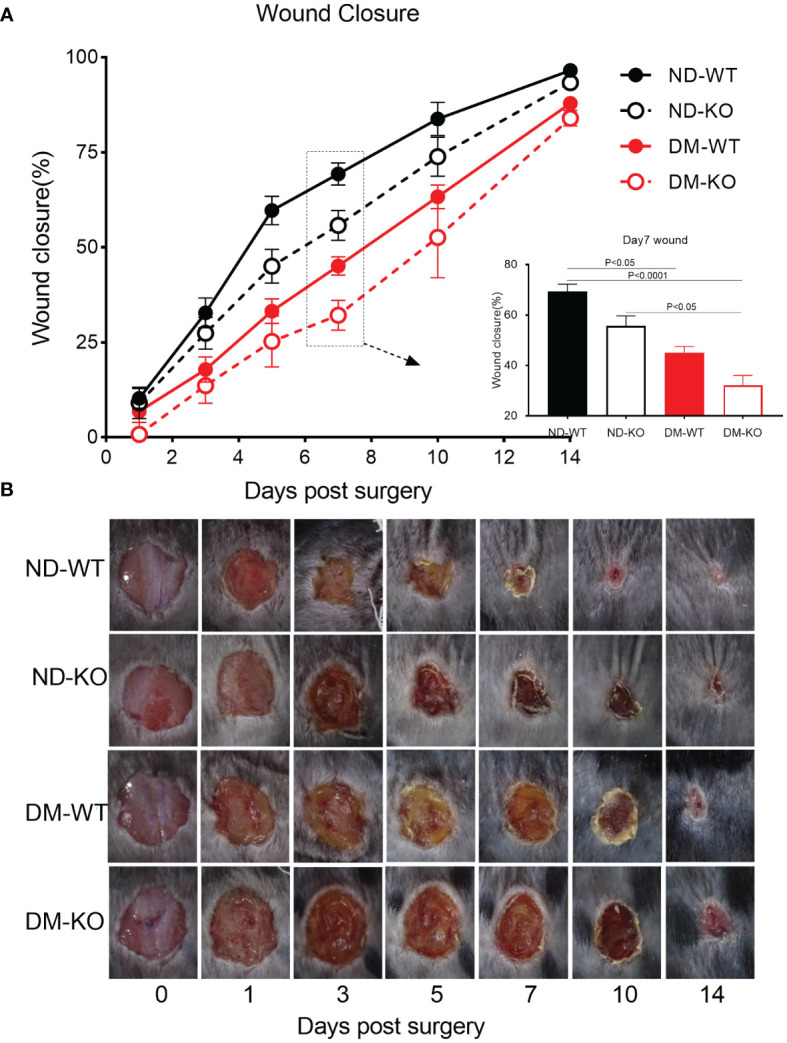
Wound closure of mice in each group. The mean ± standard deviation of wound closure rate was demonstrated **(A)**. The inset shows data for day 7. The macroscopic wound closure at the days post-surgery was showed in the photographs **(B)**.

All wounds from the four groups (non-diabetic with WT mice - ND-WT, non-diabetic with CD64 KO mice - ND-KO, diabetic with WT mice - DM-WT, and diabetic with CD64 KO mice - DM-KO) were carefully monitored and captured daily for quantification following a full-thickness skin incision that removed a 1.0×1.0 cm area of skin at macroscopic level (**Figure 3A**), which is consistent with microscopic level in HE staining (**Supplementary Figure S3**).

The three-way ANOVA showed that diabetes mellitus (P<0.0001) and CD64 KO (P<0.001) significantly influenced the wound closure. Specifically, wound closure progressed rapidly in the ND-WT mice, with almost complete closure observed on day 10 post-surgery. In contrast, wound closure in the DM-WT mice was significantly delayed, showing almost a 3-fold difference between ND-WT and DM-WT on days 1, 3, 5, and 7. However, the difference gradually reduced on days 10 and 14, although complete wound closure had not been achieved by day 14 following surgery. Consequently, wound closure in the DM-WT mice was delayed by nearly 1 week compared to the ND-WT mice. Quantitatively, wound closure was substantially impaired at all-time points in the DM-WT mice. Specifically, at days 1, 3, 5, 7, 10, and 14, wound closure in the DM-WT mice was 3.2%, 14.9%, 26.5%, 38.8%, 20.5%, and 8.6% lower than their ND-WT counterparts, respectively (**Figure 3B**). These findings highlight the considerable delay in wound healing observed in the DM-WT mice compared to the non-diabetic group with WT mice.

The wound closure in CD64 KO mice was significantly delayed compared to WT mice, particularly on days 5, 7, and 10 post-surgery. Quantitatively, at days 5, 7, and 10, wound closure in the ND-KO mice was 14.6%, 13.6%, and 9.9% lower than in their ND-WT counterparts, respectively. Furthermore, the closure in DM-KO mice was 7.9%, 13.0%, and 10.7% lower than in their DM-WT counterparts on the same respective days. These data clearly demonstrate that the loss of CD64 due to CD64 KO leads to delayed wound healing. Importantly, the extent of delayed wound healing did not differ significantly between the diabetic and non-diabetic mice groups.

In summary, the images of wound closure revealed a considerable delay in wound healing in diabetic mice compared to non-diabetic mice. Additionally, it showed a significant delay in wound healing in CD64 KO mice compared to WT mice. Moreover, there was no synergistic effect of CD64 depletion and diabetes on wound healing. These findings underscore the importance of CD64 in the wound healing process and suggest its potential therapeutic relevance in both diabetic and non-diabetic conditions.

### CD64 KO had a detrimental effect on diabetic wound healing, primarily through the reduction of CD163^+^ macrophages and TGFβ1 production

**Figure 4:**
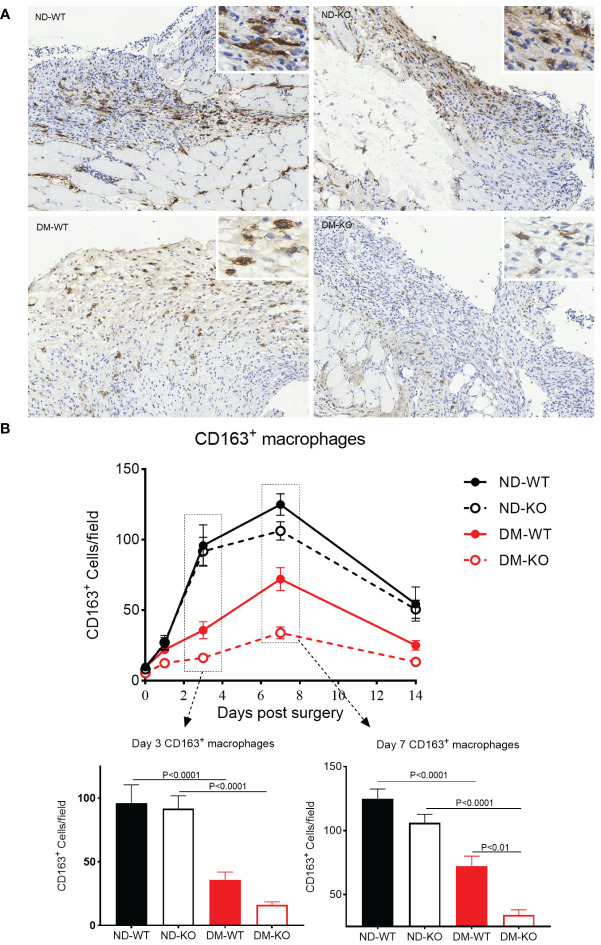
Expression of CD163^+^ macrophages in wounds across different groups detected by immunohistochemistry. CD163^+^ macrophages infiltration at the day 7 post-surgery was showed in the photomicrographs **(A)**. The mean ± standard deviation of CD163^+^ cells/filed was showed **(B)**. The inset shows data for days 3 and 7. The X-axis denotes the days post-surgery, while the Y-axis represents the expression of CD163^+^ macrophages in image units.

The three-way ANOVA showed that diabetes mellitus (p<0.0001) and CD64 KO (p<0.0001) significantly influenced the CD163^+^ macrophages. Specifically, after day 1 post-surgery, only a small number of CD163^+^ macrophages were observed in the skin in all four groups (**Figure 4A**). The infiltration of CD163^+^ macrophages gradually increased, reaching a plateau on the 7^th^ day, followed by a gradual reduction until day 14. On the 3^rd^ day after surgery, the number of CD163^+^ macrophages was higher in the non-diabetic group compared to the diabetic group. The number of CD163^+^ macrophages in ND-KO mice was similar to that in ND-WT mice, but in DM-KO mice, it was lower than in DM-WT mice, nearly twice as low on day 3. The difference between the four groups was most pronounced on day 7: ND-KO had lower CD163^+^ macrophage levels than ND-WT, and the levels in DM-KO were significantly lower than in DM-WT, being nearly four times lower than DM-KO. In summary, both the diabetic condition and CD64 gene knockout compromised wound healing, possibly through compromised inflammatory mediators and neovascularization, leading to a reduction of M2 macrophages at the wound site. However, the effect on M2 macrophages was greater in the diabetic state (**Figure 4B**).

**Figure 5:**
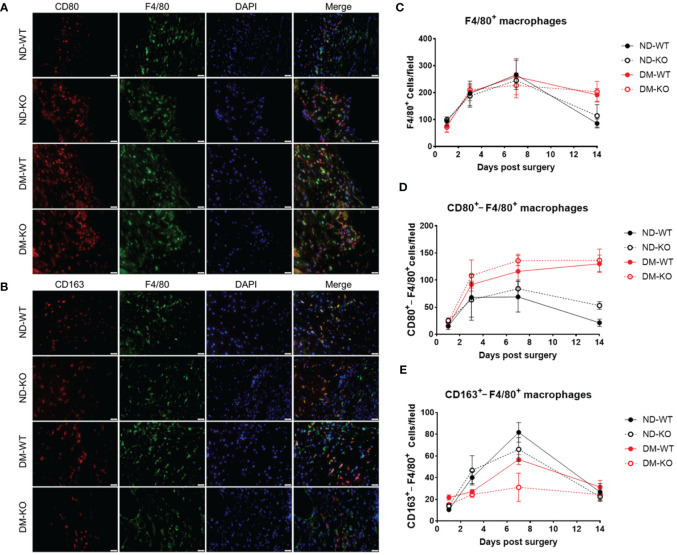
It illustrates the presence of macrophages in wounds among different groups, as detected by immunofluorescence double staining. Photomicrographs **(A)** showcase immunofluorescence staining of CD80 (in red) and F4/80 (in green) on day 7 post-surgery. Similarly, photomicrographs **(B)** display immunofluorescence staining of CD163 (in red) and F4/80 (in green) on the same day. Quantitative analysis reveals the mean ± standard deviation of F4/80^+^ cells per field **(C)**, CD80^+^ cells per field **(D)**, and CD163^+^ cells per field **(E)**. The X-axis denotes the days post-surgery, while the Y-axis represents the positive cells per field in image units.

To verify the assumption of CD163^+^ cells are M2 macrophages, we further explore the subsets of M1 and M2 macrophages, using immunofluorescent double staining, i.e. F480/CD80 (**Figure 5A**) and F480/CD163 (**Figure 5B**) labelling.

A very similar pattern of F4/80^+^ macrophages infiltration to the wounds with CD163^+^ cells was observed (**Figure 5C**). Upon stratifying F4/80^+^ macrophages into M1 (F4/80^+^/CD80^+^) and M2 (F4/80^+^/CD163^+^) cells, we demonstrated that infiltrating M1 cells were elevated on day 3, plateaued on day 7, and gradually decreased in non-diabetic ND-WT and ND-KO wounds (**Figure 5D**). Notably, infiltrating M1 macrophages exhibited a persistent increase throughout the entire wound healing period in both DM-WT and DM-KO mice.

Conversely, the infiltrating M2 macrophages in the wound were consistent with wound healing, especially under diabetic conditions (**Figure 5E**), showing that there was compromised M2 macrophage infiltration DM-WT and DM-KO mice (**Figure 5E**). It is noteworthy that there were some differences observed in infiltrating CD163^+^ cells alone and F4/80^+^/CD163^+^ M2 macrophages between CD163 single staining and CD163/F4/80 double staining in our current study (**Figures 4**, **5E**).

**Figure 6:**
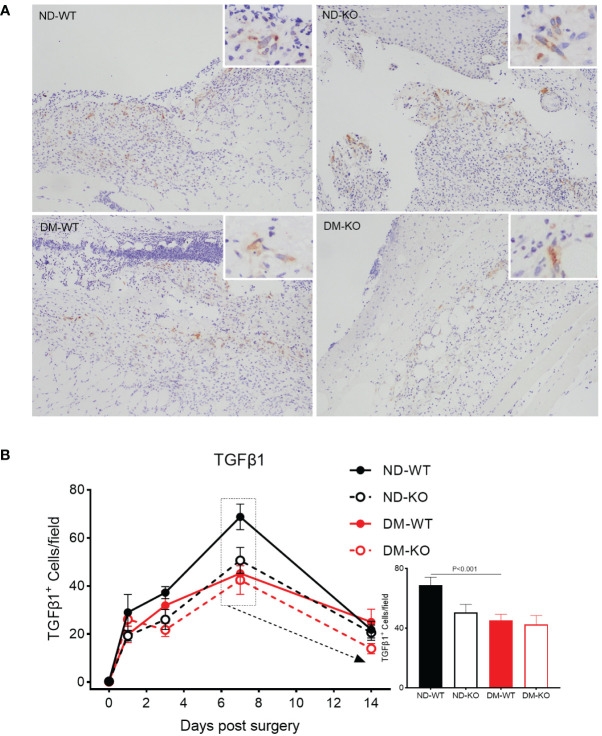
Expression of TGFβ1 in wounds across different groups detected by immunohistochemistry. TGFβ1 expression at the day 7 post-surgery was showed in the photomicrographs **(A)**. The mean ± standard deviation of TGFβ1^+^ cells/filed was showed **(B)**. The inset shows data for day 7. The X-axis denotes the days post-surgery, while the Y-axis represents the expression of TGFβ1 in image units.

Similarly, the three-way ANOVA showed that diabetes mellitus (p<0.05) and CD64 KO (p<0.01) significantly influenced the TGFβ1 production. TGFβ1 expression in the unwounded skin was almost undetectable in all four groups at day 0 (**Figure 6**). Following surgery, TGFβ1 expression increased and peaked at day 7, gradually down-regulating in all groups. However, there was a significant difference in TGFβ1 production among the groups. Notably, TGFβ1 production was highest in ND-WT, followed by ND-KO, and lowest in DM-WT, with ND-WT showing 1.5 times higher production than DM-WT (p<0.001). There were no significant differences among the other groups.

### The effect of CD64 gene knockout on angiogenesis and collagen deposition in wound healing was not significant

**Figure 7:**
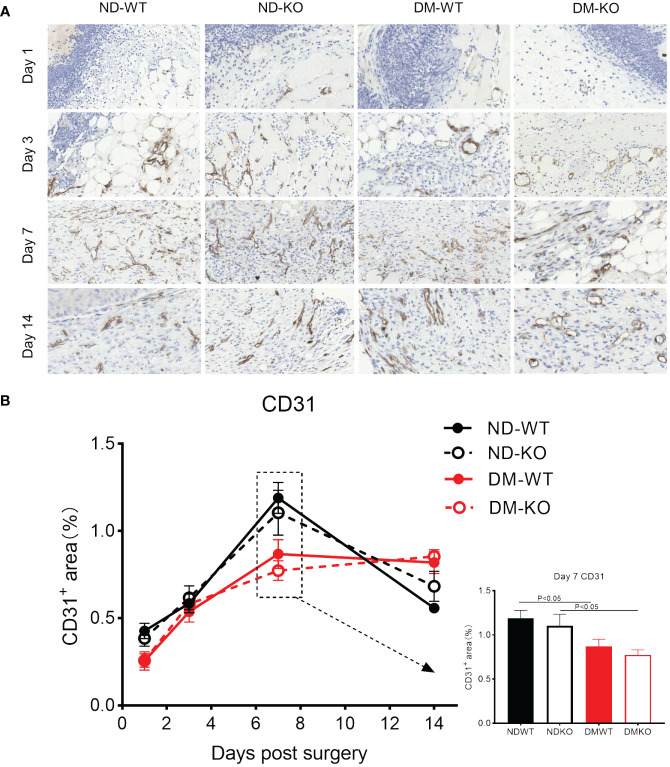
Expression of CD31 in wounds across different groups detected by immunohistochemistry. CD31^+^ microvessels at the days post-surgery was showed in the photomicrographs **(A)**. The mean ± standard deviation of CD31^+^ microvessels area rate was showed **(B)**. The inset shows data for day 7. The X-axis denotes the days post-surgery, while the Y-axis represents the area rate of CD31^+^ microvessels in image units.

Angiogenesis in the wounds was identified through CD31 immunostaining for microvessels (**Figure 7**). High-resolution images depicting CD31 labelling at higher magnification are provided (**Figure 7A**), as detailed in our previous publications (12, 25). Three-factor analysis of variance showed that diabetes had a statistical significance for angiogenesis (p<0.05), while CD64 KO had no statistical significance for angiogenesis (p=0.8787). No significant difference in microvessel number was observed among all groups in uninjured skin. Following surgery, angiogenesis in the wounds gradually increased in ND-WT, ND-KO, and DM-WT mice, peaking at day 7 (**Figure 7B**). In the DM-KO group, the area of CD31^+^ microvessels in the wounds increased steadily from day 1 to 14. A significant difference in angiogenesis was observed on day 7 between DM and non-DM groups. The area of CD31^+^ microvessels in DM-WT was lower than that in ND-WT, being ~ 1.3 times higher in ND-WT (at day 7). The area of CD31^+^ microvessels in ND-KO was nearly 1.4 times higher than in DM-KO on day 7. However, the difference between KO and WT was not significant.

**Figure 8:**
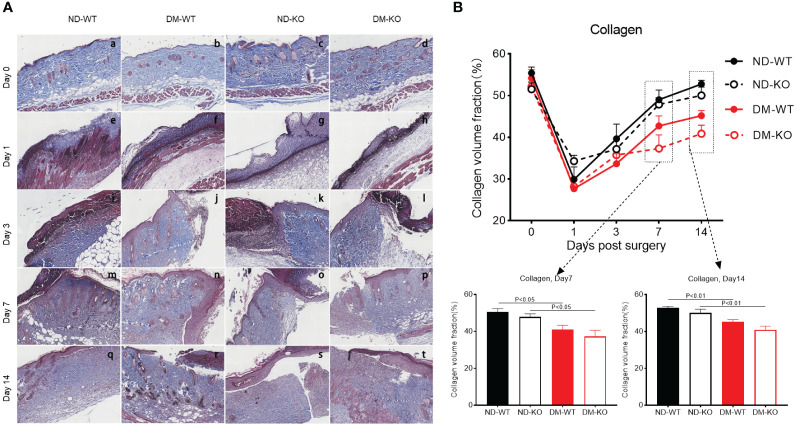
Collagen deposition in the wounds across different groups detected by Trichrome staining. The collagen deposition at the days post-surgery was showed in the photographs **(A)**. The mean ± standard deviation of CVF was showed **(B)**. The inset shows data for day 7 and 14. The X-axis denotes the days post-surgery, while the Y-axis represents the collagen volume fraction in image units.

In uninjured skin, all groups showed a collagen volume fraction (CVF) of about 55% (**Figure 8A**). Collagen deposition increased gradually in all groups following injury. There was no significant difference in collagen deposition among all groups at days 1 and 3. However, a significant difference was observed at days 7 and 14 (**Figure 8B**). Notably, collagen deposition was highest in ND-WT, followed by ND-KO, DM-WT, and DM-KO, in descending order. The difference in collagen deposition between DM and non-DM groups was statistically significant, whereas the difference between WT and KO groups was not statistically significant.

In summary, the delayed wound healing observed in diabetes correlated well with microvessel formation and collagen deposition. However, the connection between CD64 KO and microvessel formation or collagen deposition was minimal.

## Discussion

In the current study, CD64 was significantly up-regulated in the chronic ulcerative skin of DM patients compared to non-DM skin. CD64 expression correlated with FBG, GA, and HbA1c levels. Clinical observations were supported by findings in CD64 KO mice, which exhibited significant delays in wound healing, particularly in the context of diabetes. This delay was associated with compromised inflammatory mediators, emphasizing the critical role of inflammation in wound healing. However, the precise underlying mechanism of CD64 in diabetic wound healing remains to be clarified and will be further verified in clinical samples through *ex vivo* manipulation of CD64 and/or experiments *in vivo*, using animal models.

Significant delays in wound healing, particularly in diabetes, was associated with compromised inflammatory mediators, providing compelling evidence for the critical role of inflammation in the wound healing process (28). These results underscore the potential importance of CD64 in diabetic wound healing and highlight the relevance of inflammatory pathways in this complex biological process.

Wound healing involves the interplay of host immunity and inflammatory responses (5). DM patients experience considerable impairment in wound healing (29), primarily due to compromised host immunity (30). CD64, known to be up-regulated on neutrophils in various conditions such as septicemia (31), neonatal severe respiratory infection (32) and septic shock (33), plays a role in host defense against microbial invasion (34).

While compromised wound healing was evident in CD64 KO mice, the potential influence of wound contraction on the observed delayed closure cannot be conclusively ruled out. Future experiments employing splinting techniques will further investigate its impact on the wound healing process, correlating with TGFβ1 expression. To address the possibility of delayed wound closure being attributed to wound contraction, upcoming experiments using splinting techniques will confirm its impact on the healing process (35).

Our previous research demonstrated substantial impairment in wound healing in GM-CSF depleted animals with DM (24), accompanied by reduced recruitment of neutrophils and macrophages (12). However, the deficiency in wound healing in DM could be restored with exogenous GM-CSF, along with increased pro-inflammatory cytokines (12). These findings underscore the crucial role of inflammation in acute wound healing, and any impairment in inflammatory responses may lead to delayed wound healing (36). Understanding the intricate interplay between host immunity, inflammation, and wound healing in the context of diabetes could offer valuable insights for potential therapeutic interventions in improving wound healing outcomes for DM patients.

Considering CD64 is involved in host inflammatory responses during acute inflammation (14) and its association with certain autoimmunities (37), it is reasonable to speculate that CD64 may negatively impact wound healing via dysregulating local host immunity in DM skin. This speculation aligns with our current finding of substantially increased CD64 expression in the dermal layer of DM chronic ulcerative skin compared to non-DM skin. The upregulated expression of CD64 might be compensating for compromised host immunity in diabetic wounds, resulting in delayed wound healing. This observation is consistent with the up-regulation of inflammatory mediators, such as IL-1β, TNF, and IL-8, in DM skin, further supporting the potential role of CD64 in the wound healing process within chronic ulcerative skin.

To validate the role of CD64 in diabetic wound healing, we found a significant reduction in wound healing in CD64 KO mice compared to WT mice, especially under diabetic conditions. Such findings highlight the essential role of CD64 in the diabetic wound healing process, consistent with the previously observed compromised host immunity (38). Notably, there wasn’t a synergistic effect between CD64 expression and the DM condition in this acute wound healing model. This could be attributed to the short duration of the current study, which may limit the impact of long-term host immunity disturbances. Therefore, further investigation is needed to explore the correlation between CD64 and long-term DM in wound healing and its underlying mechanisms.

Nevertheless, our data may offer valuable insights for managing chronic ulceration in DM patients, using CD64 as a potential therapeutic target. Furthermore, it encourages further research to better understand the complexities of wound healing in diabetes and its association with CD64 mediated inflammatory responses. However, we recognize that the current wound healing in the diabetic animal model can’t fully reflect the clinical chronic wounds and/or ulcerations in patients with diabetes mellitus due to differences in species and disease progression (weeks in animal models versus years in humans).

Previous studies identified characteristic markers for M1 and M2 cells, including CD80, CD86, CD38, HLA-DR for M1, and CD163, CD206, CD204, MerTK for M2 (39). Our earlier publications emphasized the role of mature macrophages in diabetic wound healing (12, 24), without distinguishing between M1 and M2. CD163^+^ M2 macrophages contribute significantly to wound healing by promoting anti-inflammatory responses and releasing IL-10 and IL-35 (40). Thus, we used the CD163 biomarker to explore mechanisms of diabetic wound healing.

CD163^+^ macrophages, representing the main subset of polarized M2 macrophages, play a pivotal role in wound healing by producing elevated levels of anti-inflammatory cytokines and growth factors, including IL-10, IL-1β, and TGFβ. These macrophages are associated with critical wound healing processes such as angiogenesis, matrix maturation, phagocytosis, and anti-inflammatory effects, collectively accelerating wound healing (41–44). Results revealed a reduction in CD163^+^ macrophages, particularly in DM mice with CD64 depletion, suggesting impaired diabetic wound healing by decreasing CD163^+^ M2 macrophages, possibly *via* active cytokine secretion, including TGFβ1 (45). This aligns with the gradual upregulation of TGFβ1 in all groups, more significantly in the ND-WT group. Further investigations are warranted to elucidate these interactions and their impact on wound healing in diabetics.

Verification of M1 and M2 macrophage infiltration suggest that M1 may play a crucial role in chronic wound clearance, particularly under diabetic conditions, this is supported by the finding that high glucose impairs keratinocyte migration via inducing M1 macrophage polarization (46). Such finding is also aligned with our macroscopic findings, showing substantial compromised wound healing *in vivo*. It is noteworthy that there were some differences observed in infiltrating CD163^+^ cells alone and CD163^+^/F4/80^+^ M2 macrophages between CD163 single staining and CD163/F4/80 double staining in our current study. This discrepancy could be attributed to variations in sensitivity between immunohistochemical staining and immunofluorescent staining. Additionally, it is important

to acknowledge that not all CD163^+^ cells necessarily represent macrophages, e.g. dendritic cells may also express CD163 (47), and we plan to verify this point in future studies.

Neo-vascularization increased gradually, peaking at day 7, aligning with our previous publications (12, 24), supporting the importance of neo-vascularization in wound healing. The area of neo-vascularization was highest in ND-WT, followed by ND-KO, DM WT, and DM-KO, consistent with macroscopic observations, suggesting that neo-vascularization enhances wound healing.

In DM, wound healing is suppressed, inhibiting wound closure by suppressing neutrophil and macrophage recruitment, delaying fibroblast proliferation, and collagen secretion (30). Our findings demonstrate delayed wound healing in CD64 KO mice, particularly in the presence of DM, suggesting a role for CD64 expressed neutrophils in wound healing (14). Additionally, we observed reduced fibrosis in DM-KO mice but increased inflammation and vascular congestion. The delayed wound healing in diabetes correlates with collagen deposition, but the connection between CD64 depletion and collagen deposition appears minimal, warranting further investigation.

Initially, we planned to utilize 8-12 week-old mice to synchronize and minimize the effects of epithelial hair follicle stem cells, and to investigate the subsequent contribution of anagen hair follicles to wound healing. However, our plan experienced significant delays due to a mandatory lockdown for two months in Shanghai, triggered by the Omicron variant of the SARS-CoV-2 viral outbreak (48). As a result, we had to use 14-week-old mice, facing financial, staffing, and ethical challenges in discarding the original batch of animals. Using 14-week-old mice may introduce issues related to epithelial hair follicle stem cells, yet this study serves as a proof of concept, highlighting the significant role of CD64 in diabetic wound healing. Future research will explore other contributing factors. To mitigate potential genetic effects on wound healing, we employed CD64 knockout (ko) mice, which underwent over six generations of backcrossing with C57B/6 mice. Moving forward, we plan to conduct the study using het and het crosses to further minimize such potential influences.

## Conclusions

In conclusion, our study highlights the significant role of CD64 in wound healing, particularly in the context of diabetes. The correlation between CD163^+^ macrophages and wound healing indicates that CD64 is closely linked to host immunity during the wound healing process. These findings hold promise for providing valuable insights to clinicians in the management of diabetic chronic ulceration. Understanding the intricate interplay between CD64, host immunity, and wound healing pathways may pave the way for potential therapeutic strategies to improve wound healing outcomes in diabetic patients.

## Data availability statement

The original contributions presented in the study are included in the article/**Supplementary Material**. Further inquiries can be directed to the corresponding authors.

## Ethics statement

The studies involving humans were approved by Ethics Committee, Tongren Hospital, Shanghai Jiaotong University School of Medicine. The studies were conducted in accordance with the local legislation and institutional requirements. The participants provided their written informed consent to participate in this study.

## Author contributions

XZ: Investigation, Data curation, Project administration, Writing – original draft. ZT: Writing – review & editing, Investigation, Methodology. HW: Writing – review & editing. FC: Writing – review & editing. LY: Writing – review & editing. JH: Writing – original draft. PW: Writing – review & editing. HB: Writing – review & editing. SB: Conceptualization, Writing – review & editing. TK: Conceptualization, Formal analysis, Funding acquisition, Writing – review & editing.

## Supplementary material

Supplementary Figure S1 Schematic diagram illustrating PCR amplification for the identification of Wt or CD64 KO mice.

Supplementary Figure S2 PCR confirmation of CD64 KO mice, with the top band exclusively representing CD64 KO mice, the bottom band exclusively indicating Wt mice, and double bands indicative of Het mice. The red circles represent CD64 KO male mice.

Supplementary Figure S3 Hematoxylin and eosin staining of wound tissues at the days post-surgery.
