# Supplementary figures and images for: CD64 plays a key role in diabetic wound healing
Source: Front Immunol. 2024 Mar 8;15:1322256. doi: 10.3389/fimmu.2024.1322256 (PMC10957625; doi:10.3389/fimmu.2024.1322256)

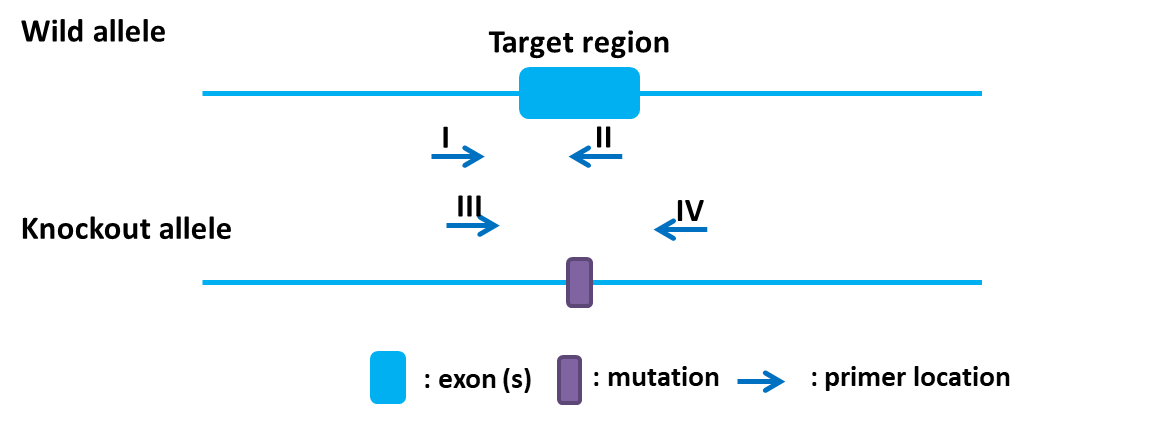

Supplement: Supplementary Figure S1 — Schematic diagram illustrating PCR amplification for the identification of Wt or CD64 KO mice. [file Image_1.tif]

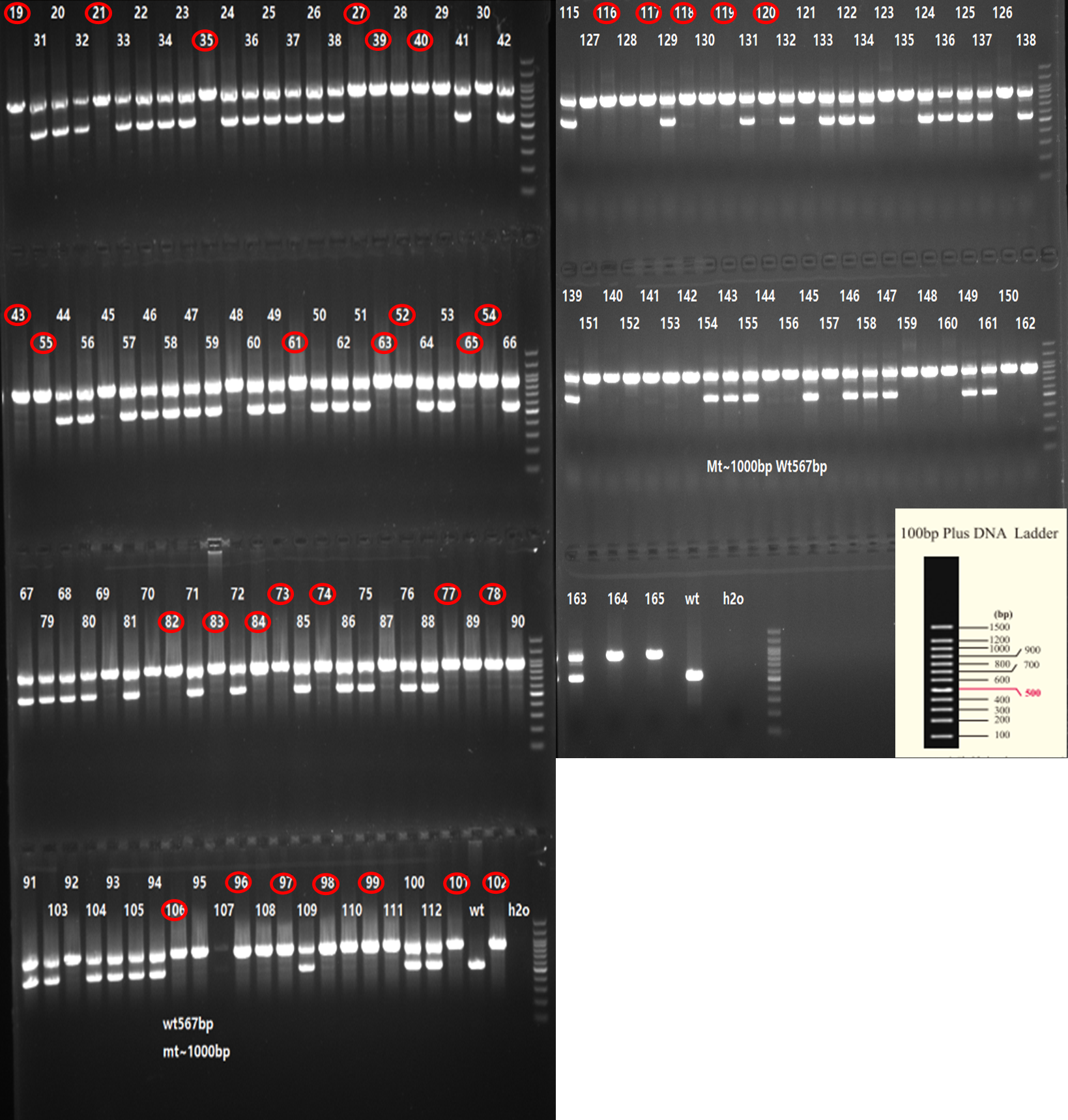

Supplement: Supplementary Figure S2 — PCR confirmation of CD64 KO mice, with the top band exclusively representing CD64 KO mice, the bottom band exclusively indicating Wt mice, and double bands indicative of Het mice. The red circles represent CD64 KO male mice. [file Image_2.tif]

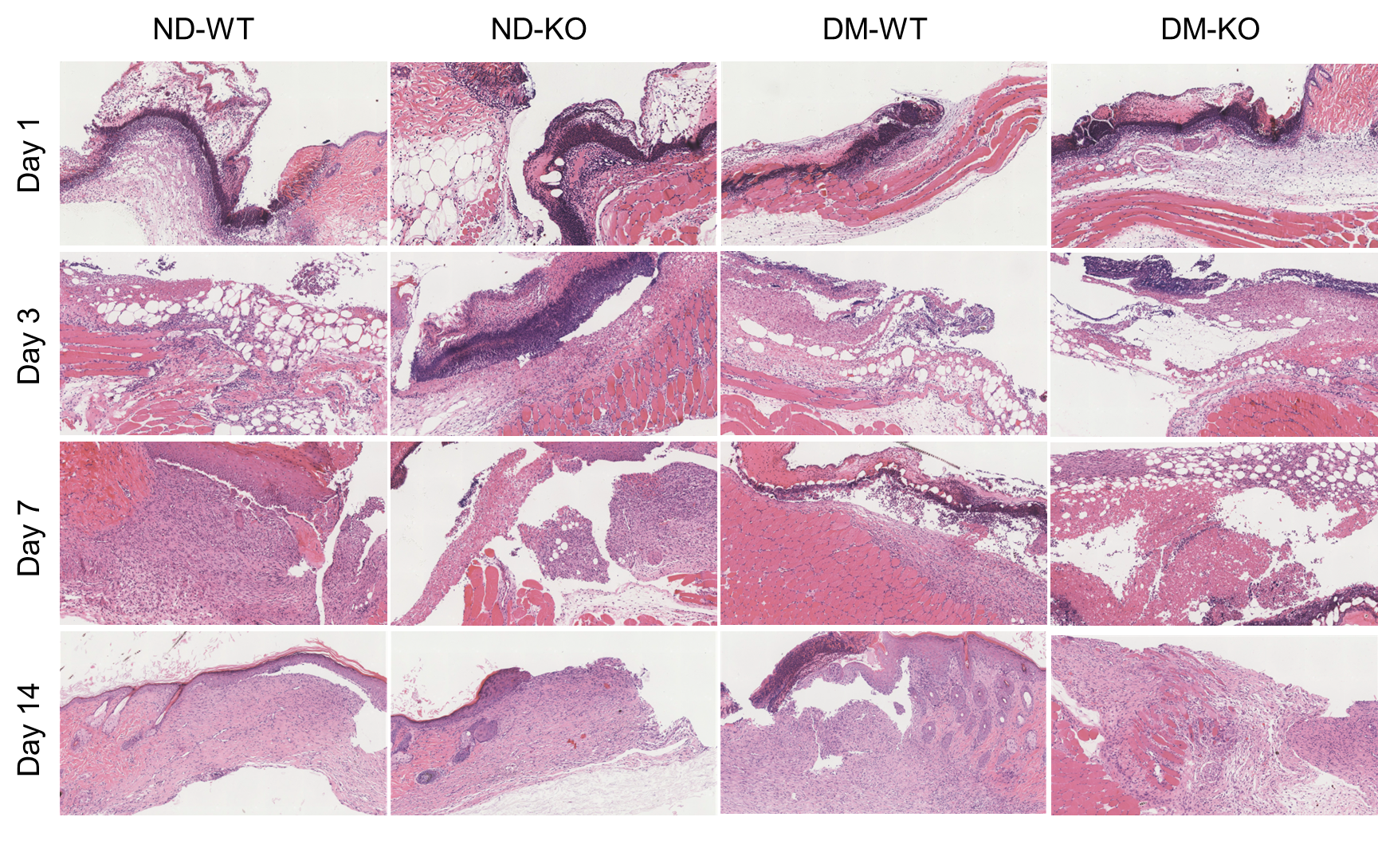

Supplement: Supplementary Figure S3 — Hematoxylin and eosin staining of wound tissues at the days post-surgery. [file Image_3.tif]
